# An Outbred Calf Model for Determining Innate Immune Sensing and Evolutionary Trajectories of a Cell Culture-Adapted Bovine Foamy Virus Variant

**DOI:** 10.3390/v15081772

**Published:** 2023-08-20

**Authors:** Magdalena Materniak-Kornas, Piotr Kubiś, Bartosz Sell, Georgios Pougialis, Martin Löchelt, Jacek Kuźmak

**Affiliations:** 1 Department of Biochemistry, National Veterinary Research Institute, 24-100 Pulawy, Poland; kubip@piwet.pulawy.pl (P.K.); jkuzmak@piwet.pulawy.pl (J.K.); 2 Department of Pharmacology and Toxicology, National Veterinary Research Institute, 24-100 Pulawy, Poland; bartosz.sell@piwet.pulawy.pl; 3 Division of Viral Transformation Mechanisms, Research Program Infection, Inflammation and Cancer, German Cancer Research Center, 69120 Heidelberg, Germany; g.pougialis@dkfz.de (G.P.); m.loechelt@dkfz.de (M.L.); 4 Division of Virus-Associated Carcinogenesis, Research Program Infection, Inflammation and Cancer, German Cancer Research Center, 69120 Heidelberg, Germany

**Keywords:** bovine foamy virus, cell-free and cell-associated variants, replication *in vivo*, calves

## Abstract

Bovine foamy virus (BFVbta) displays a very high degree of cell-associated replication which is unprecedented even among the other known foamy viruses. Interestingly, recent studies have shown that it can in fact adapt *in vitro* to high-titer (HT) cell-free transmission due to genetic changes acquired during repeated rounds of cell-free BFVbta passages in immortalized bovine MDBK cells. Molecular clones obtained from the HT BFVbta Riems cell-free variant (HT BFVbta Riems) have been thoroughly characterized in MDBK cell cultures However, during recent years, it has become increasingly clear that the source of the host cells used for virus growth and functional studies of virus replication and virus–cell interactions plays a paramount role. Established cell lines, mostly derived from tumors, but occasionally experimentally immortalized and transformed, frequently display aberrant features relating, for example. to growth, metabolism, and genetics. Even state-of-the-art organoid cultures of primary cells cannot replicate the conditions in an authentic host, especially those concerning cell diversity and the role of innate and adaptive immunity. Therefore, to determine the overall replication characteristics of the cloned wt and HT BFVbta Riems variant, we conducted a small-scale animal pilot study. The replication of the original wt BFVbta Riems isolate, as well as that of its HT variant, were analyzed. Both BFVbta variants established infection in calves, with proviruses in peripheral blood mononuclear cells and induced Gag-specific antibodies. In addition, a related pattern in the host innate immune reaction was detected in the peripheral blood leukocytes of the BFV-infected calves. Surprisingly, an analysis of the Gag sequence two weeks post-inoculation revealed that the HT BFVbta variant showed a very high level of genetic reversion to the wild type (parental BFVbta genotype).

## 1. Introduction

Bovine foamy virus (BFVbta, also known as BFV) is a retrovirus of the subfamily *Spumaretrovirinae* which is characterized by substantial differences in its molecular biology when compared with other retroviruses [1,2,3]. BFVbta has a high prevalence in domestic cattle, but has not been correlated with any clinically relevant bovine disease (summarized in [2]). *In vivo*, BFVbta target cells are ill-defined, but they include leukocytes, which may be the reason why BFVbta can be isolated from or detected in different lymphoid organs as well as in the lungs, milk, and liver [2]. All known BFVbta isolates are highly cell-associated *in vitro*, and natural transmission to calves may be mediated via milk containing BFVbta-infected leukocytes, but not via cell-free virus particles [4,5,6], unlike some other retroviruses, such as human T-cell leukemia virus (HTLV) [7], mouse mammary tumor virus (MMTV) [8], and bovine leukemia virus (BLV) [9].

Since serum levels of antibodies towards the structural Gag and the accessory Bet protein are high in infected animals of different age groups, it is highly probable that continuous or at least sporadic expression of viral proteins occurs during BFVbta persistence or latency in the host [6]. The degree, relevance, and underlying mechanism(s) of BFVbta latency or persistence in cell cultures, and especially in infected animals, are not currently known.

To address questions concerning cell-free transmission versus cell–cell transmission and virus persistence and latency, we and others have exploited the very tight cell association of BFVbta and its lack of cell-free transmission for mechanistic studies related to this special feature in cell cultures [1,10,11]. As an infectious agent in livestock animals, BFVbta is suitable for animal experiments in the authentic host species, and such experiments may be used to supplement *in vitro* studies in more or less artificial, simplified, and complexity-lacking surrogate models [12,13,14]. The BFVbta Riems isolate used in this and our previous studies had been solely propagated in primary bovine trachea cells (KTR cells) using co-cultivation techniques [10,15]. The parental BFVbta Riems isolate from KTR cells was sequenced [16], and molecular clones were constructed [17]. Following transfection into human HEK293 cells and the subsequent infection of fully permissive bovine Madin-Darby Bovine Kidney (MDBK) cells, these clones displayed cell-associated replication indistinguishable from that of uncloned BFVbta virus stocks [1,17]. To identify the mechanisms of the tight cell association, a stock of the original BFVbta Riems virus was subjected to *in vitro* selection and evolution assays in order to gain variants capable of cell-free high-titer (CF-HT) transmission *in vitro* using established (immortalized) bovine MDBK and baby hamster kidney (BHK-21) cells [10]. *In vitro* functional analyses confirmed cell-free HT transmission that was not due to immune escape mechanisms [10]. In addition, bioinformatics of sequences obtained during the adaptation process revealed shared and cell type-specific structural changes in the Gag and Env proteins, the principal assembly and budding machinery of FVs and retroviruses in general [1,10,18]. The gag-pol-env region of the selected variant was cloned into the parental pBFV-Riems backbone, producing BFVbta variants capable of cell-free HT transmission. Evolutionary analyses showed that the acquisition of an *in vitro* cell-free HT transmission phenotype would require relatively few amino acid exchanges, and that these would initially affect Env and then Gag. Since in FVs, as in all other retroviruses, Gag and Env are major players in particle-associated infectivity [18], there is a strong dependency on changes in both partners, and some changes in Gag and/or Env appear to be linked to each other [1]. Finally, cell type-specific and random stochastic processes lead to CF-HT BFVbta variants with relatively few (though consistent) adaptive changes in both genes [1]. Similar findings have also been described for another BFV isolates [11].

The dynamics and success of the virus–host interaction with respect to virus reproduction depends not only on the speed of virus production and release and the infection of new host cells mainly encoded by the canonical retroviral Gag, Pol, and Env proteins, but also on accessory viral proteins and regulatory elements. Several of these additional genes are only needed in defined cell types, and thus comprise accessory genes not absolutely essential for basic virus production [19,20], although their products target defined host cell proteins that are present within the cellular pathway needed for virus replication or that belong to the intrinsic, innate, or adaptive immunity network responsible for restricting virus infection, replication, and spread, and which are involved in virus clearance [21].

Here, we described a small-scale animal study undertaken to determine the potential differences in the overall replication characteristics of the cloned wt and HT BFVbta Riems variants and, in addition, the host-directed innate and adaptive immune responses to these viruses. The data obtained also show that the cloned BFVbta Riems isolate is replication competent in calves, and this enables the *in vivo* testing of engineered variants and reverse genetic studies in the authentic host. However, it appears that the *in vitro*-selected HT BFVbta Riems variant almost always reverts to its parental genotype, retaining few newly acquired changes, and this may be relevant for virus replication in the natural, outbred host.

## 2. Materials and Methods

### 2.1. Animals and Experimental Inoculations

All animals used in this study were purchased from local suppliers and adapted to the housing and feeding conditions in the animal facilities of the National Veterinary Research Institute in Pulawy, Poland, before the beginning of the study. The study was approved by the local ethical commission and was conducted in accordance with the national regulations for animal experimentation (permit no. 59/2017). Six 6-month-old Holstein Friesian calves which tested seronegative for BFVbta, bovine leukaemia virus (BLV), and bovine viral diarrhoea virus (BVDV) were used. The calves were divided into three groups of two calves. The animals were inoculated intravenously with 2.5 × 10^6^ MDBK (Madin Darby Bovine Kidney) cells infected with the respective BFVbta variants and 20 mL of cell culture supernatant. The first group was inoculated with a high-titer BFVbta variant (pCMV-BFVbta-MDBK-24, sub-clone wt24; HT BFVbta Riems), and the second group was inoculated with a BFVbta Riems isolate (pCMV-BFVbta-Riems). Briefly put, the BFV Riems variants and the uninfected cells were cultivated in MEM with Earl’s salts medium supplemented with 10% horse serum and 1x antibiotic-antimycotic (Sigma-Aldrich, St. Louis, MO, USA). The presence of similar levels of BFVbta replication as well as BFVbta protein expression was confirmed using indirect immunofluorescence in all of the cell cultures used in the experiment. Between 50 and 60% of the cells expressed the BFVbta antigen in the time immediately preceding the collection of cells and the preparation of the inoculum. The third group was the mock-infected control group; this group was inoculated with 2.5 × 10^6^ uninfected MDBK cells and 20 mL of supernatant. Throughout the experiment, each group of calves was kept isolated from the others. Blood samples were collected from the jugular vein of each animal and added to tubes containing anticoagulant (EDTA-Na) both before inoculation (t0) and on each of the 6 days following inoculation. Later, starting in the second week, blood was collected every second week until week 16 post-inoculation (p.i.).

Peripheral blood mononuclear cells (PBMCs) were isolated via gradient density centrifugation using Histopaque 1077 (Sigma-Aldrich, St. Louis, MO, USA), frozen, and stored at −70 °C until DNA and RNA purification [22]. Peripheral blood leukocytes (PBLs) were obtained using the haemolysis method as described in [13]. Blood was haemolysed with ice-cold H_2_O and 4% NaCl, and then centrifuged. The PBLs were washed twice with PBS, divided into aliquots of 5 × 10^6^ cells, and used directly for co-cultivation with MDBK cells for virus recovery or frozen at −70 °C until further processing.

### 2.2. Antibody Detection

A GST-capture ELISA was used to examine the antibody response to the BFVbta antigen in the sera of the experimentally infected calves, as described in [6]. In brief, all of the serum samples were tested in duplicate for GST and GST-Gag fusion proteins as antigens. Prior to their application to the plates, all the serum samples were additionally incubated with the lysate of a GST-expressing *E. coli* culture (2 μg/μL) so that they would pre-absorb GST-binding antibodies. Protein G peroxidase conjugate (Sigma-Aldrich, St. Louis, MO, USA, 1:5000) was used as a secondary antibody, and tetramethylbenzidine (TMB, Sigma-Aldrich, St. Louis, MO, USA) (0.1 mg per 1 mL of acetate buffer) was added as a substrate. The specific reactivity against the BFVbta antigen (net absorbance value) was calculated for each serum sample by subtracting the absorbance measured for the GST from the absorbance noted for the GST-Gag protein (this is presented as an average value from two replicates).

### 2.3. Determination of BFVbta Viral DNA Load in Peripheral Blood Mononuclear Cells Using qPCR

Total DNA was extracted from 5 × 10^6^ PBMCs from the experimentally inoculated calves using a Blood & Tissue Kit (Macherey-Nagel, Düren, Germany) in accordance with the manufacturer’s instructions. DNA concentrations were measured spectrophotometrically using Nanophotometer Pearl (Implen, München, Germany) and stored at −20 °C until they were used.

Two independent qPCRs were performed using the same sample to measure the BFVbta *env* and gapdh copy numbers. For this purpose, two sets of primers and probes for qPCR were designed (Genomed, Warsaw, Poland): one was to match 100% the *env* gene from the full-length BFVbta isolates (GenBank: U94514, AY134750.1, JX307861, JX307862) (BFVe7010F: AAGCCTGTTCAGGTGAATTT; e7109R: GTGAAAGTCCCAATACCCTCTC; BFVe7037P: IRD700-ATACCACAGGGCTTGTTCCTGGAA-BHQ3), and the other was to be specific for the bovine *gapdh* gene (exBTG6F: CTCACTTGAAGGGTGGCGC; BTG9R: TGACAATCTTGAGGGTGTTGTT; exBTG8P: JOE-ATCATCTCTGCACCTTCTGCCGAT-BHQ1). Both reactions were performed in duplicate using the QuantiTect Probe qPCR Kit (Qiagen, Hilden, Germany) as recommended, with the addition of 0.4 μM of each primer, 0.2 μM of probe specific for BFVbta *env*, 0.2 μM of each primer, and 0.1 μM of probe specific for *gapdh*. DNA- and PCR-grade water was added to a final volume of 20 μL. The reactions were carried out in a Rotor-Gene Q (Qiagen, Hilden, Germany) under the following conditions: initial incubation and polymerase activation at 95 °C for 15 min; denaturation at 95 °C for 15 sec; and annealing/elongation at 60 °C for 1 min for 45 cycles. Standard curves were generated in each run using 10-fold dilutions of plasmid DNA (5 × 10^2^ to 5 × 10^6^) containing either the whole amplicon of BFVbta *env* (pCR2.1/BFV) or bovine *gapdh* (pJet1.2/gapdh). Based on both reactions, the number of BFVbta copies in each sample was calculated per 1 × 10^6^ PBMCs.

### 2.4. Virus Re-Isolation

For virus re-isolation, a total of 5 × 10^6^ peripheral blood leukocytes (PBLs) from the inoculated calves were co-cultivated with 5 × 10^5^ BFVbta-free MDBK cells, which were maintained as described above (Section 2.1) in 25 cm^2^ flasks. The non-adherent cells were removed after 24 h, and the cells were cultivated for three passages. Since the high-titer BFVBTA Riems variants do not induce syncytia [10], indirect immunofluorescence (IIF) was performed using a BFVbta Gag-specific antiserum to confirm the presence and replication of the BFVbta as follows: cells grown in six-well plates were fixed in ice-cold 100% methanol and 0.02% EGTA and frozen until immuno-staining at −20 °C. Before IIF, the cells were thawed at RT, incubated for 5 min in PBS for rehydratation, and permeabilized in PBS with 0.2% Triton-100 for 20 min. A polyclonal rabbit immune serum specific for recombinant BFVbta Gag-MA (1:500 in PBS containing 3% BSA) was used as the primary antibody. Following 1 h incubation at 37 °C, the cells were washed three times for 10 min in 0.1% Tween-20 in PBS and incubated with goat anti-rabbit AlexaFluor 594 secondary antibody (Life Technologies Europe, Bleiswijk, The Netherlands) diluted 1:1000 in PBS with 1.5% BSA. For nuclear staining, Hoechst 33342 (Sigma-Aldrich, St. Louis, MO, USA) was added at a 1:1500 dilution. Cells were incubated 1 h in 37 °C, washed twice in 0.1% Tween-20 for 10 min and once in PBS for 10 min, and examined for BFVbta Gag-specific immunofluorescence using a Zeiss Observer.D1 fluorescent microscope (Carl Zeiss Microimaging, Göttingen, Germany).

### 2.5. Analysis of Gag Gene Sequence

The full-length *gag* gene of BFVBTAbta was amplified from the leukocytes of the calves two weeks post-inoculation and from the inoculum of the HT variant with following primers: Gag _S (AATGGCTCTTAATGACTTCGACCCTATAGC) and Gag_AS (AGATGATTGCCCTTGATTTCCACTTGAAGTGG). A PCR was performed in a TAdvanced thermocycler (Analytic Jena, Göttingen, Germany) under following conditions: 35 cycles of 98 °C for 10 s and 68 °C for 60 s with an additional final polymerization step of 68 °C for 5 min. The PCR reaction mixture consisted of 1 × PrimeStar PCR Premix with PrimeSTAR Max DNA Polymerase (TAKARA BIO Inc., Kusatsu, Japan) and 0.4 μM of each primer, and 0.5 μg of genomic DNA was used as the template. The expected size of the amplicon was 1633 bp. The PCR products amplified from calves no. 1 and 2 and the inoculum were cloned into pDRIVE vector (Qiagen, Hilden, Germany), and positive clones were selected. The PCR products from calves no. 3 and 4 were amplified in five independent reactions and pooled. Four positive clones from calves no. 1 and 2 and the inoculum (HT) as well as the pooled PCR products from calves no. 3 and 4 were sequenced using the Sanger method in both directions (Genomed, Warsaw Poland). The Gag consensus sequences obtained from calves and the inoculum were translated into amino acid sequences and aligned with BFVbta references using a Geneious alignment module and Geneious Pro 5.3 software (Biomatters Ltd., Auckland, New Zealand). In order to perform evolutionary analyses, the alignment was submitted to the MEGA 6.0 version [23] so that the best model could be selected based on the Bayesian information criterion (BIC) and the corrected Akaike information criterion (AIC). In accordance with the results, the Kimura-2-parameter model [24] was applied to infer a phylogenetic tree using the maximum likelihood method. The statistical confidence limits of the phylogram topologies were assessed using 1000 bootstrap replicates.

### 2.6. Relative Gene Expression Analysis of Bovine PBMCs

A relative gene expression analysis was performed using Custom RT^2^ Profiler PCR Arrays (Qiagen, Germantown, MD, USA) for the panel of genes involved in antiviral immune response. Total RNA was extracted from the PBMCs using a Nucleospin RNA Plus Extraction kit (Macherey-Nagel, Düren, Germany). RNA concentrations were determined using a Nanophotometer, and RNA integrity was assessed using an Agilent 2100 Bioanalyzer (Agilent Technologies, Waldbronn, Germany). cDNA was synthesized from 0.5 µg of RNA using an RT^2^ First Strand Kit (Qiagen, Germantown, MD, USA) in accordance with the manufacturer’s instructions, which included a genomic DNA elimination step. mRNA profiling studies were performed using custom-made RT^2^ Profiler PCR Arrays (CLAB22988, Qiagen, Germantown, MD, USA) and RT^2^ SYBR Green Mastermix (Qiagen, Germantown, MD, USA) in an ABI 7500 Real Time PCR System (ThermoFisher Scientific, Foster City, CA, USA) in accordance with the manufacturer’s instructions. Each sample was examined in duplicate. An analysis of the PCR array data was carried out in accordance with the manufacturer’s instructions using a Microsoft Excel macro available from the manufacturer (http://pcrdataanalysis.sabiosciences.com/pcr/arrayanalysis.php, accessed on 6 November 2019). Each array contained four housekeeping genes (Actb, Gapdh, PGK1, and Hsp90ab1). However, only Actb expression fulfilled the criteria for sample normalization. The transcript level of each candidate gene was quantified according to the ΔΔCt method. Ct values > 35 were not included in the analysis and considered negative. The genes were determined to be differentially expressed if the fold change (FC) was greater than 2 or lower than 0.5.

The protein interaction network analysis and its visualization were performed using the STRING Pathways database (v.12.0) for *Bos taurus* [25]. The analyses were performed under the following basic settings: full STRING network; meaning of network edges: confidence; active interaction sources: text-mining, experiments, databases, co-expression, gene fusion, neighborhood, and co-occurrence; minimum required interaction score: medium (0.400); max number of interactors to show: none. The false discovery rates (FDRs) were calculated using the Benjamini–Hochberg procedure and are shown as *p*-values corrected for multiple testing within each category. This measure describes how significant the enrichment is.

### 2.7. Interleukin 6 and Interferon Concentrations in the Claf Plasma

The interleukin 6 (IL-6) concentrations in the calf plasma samples were determined using a Bovine IL-6 High-Sensitive ELISA Kit (Cloud-Clone Corp., Huston, TX, USA). The secretion of type I interferons was tested using a CLIA Kit for Bovine Interferon Alpha (Cloud-Clone Corp., Huston, TX, USA) for interferon alpha (IFN-α) and a Nori Bovine IFN-β ELISA Kit (Genorise Scientific, Inc., Glen Mills, PA, USA) for interferon beta. All tests were performed in accordance with the manufacturer’s specifications. The plasma samples were diluted as recommended by the manufacturers in the diluents included in the respective assays and tested in duplicate. The concentration was calculated on the basis of a standard curve determined using CurveExpert Basic software.ver 1.4.

## 3. Results

### 3.1. Replication Kinetics and Characteristics of Different BFVbta Riems Variants in Experimentally Inoculated Calves

A total of six Holstein Friesian calves aged six months and known to be free of BFVbta, BLV, and BVDV pathogens were injected with cells and cell culture supernatants from mock-infected, wt BFVbta Riems-infected, and HT-BFVbta-infected MDBK cells (two animals per group) and studied for 16 weeks. Firstly, infection with cell-associated BFVbta and cell culture supernatant was performed because this method has been previously established for the Polish BFVbta100 isolate [12] and because wt BFVbta Riems is either not released or is only minimally released by MDBK cells [1,10]. Secondly, only two animals per group were used since the aim of this pilot study was to determine whether the cloned wt and HT cell free BFVbta Riems variants replicate in immuno-competent outbred calves.

**Figure 1:**
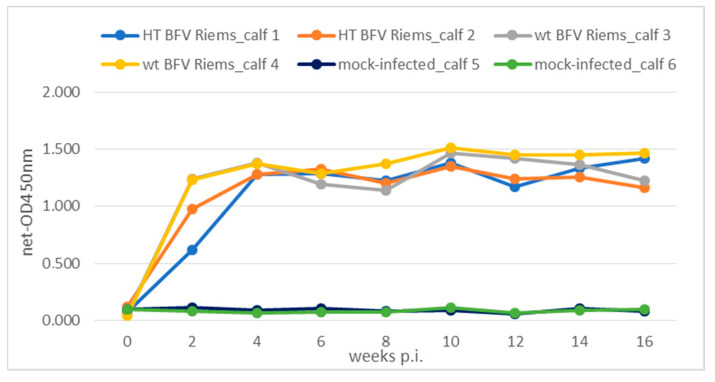
Sero-reactivity of the individual calves to BFVbta Gag following inoculation with different BFVbta Riems variants over time. The humoral response to BFVbta antigens was tested using a GST-ELISA at 16 wk p.i. in the four calves experimentally inoculated with the different variants of BFVbta Riems (calves 1 and 2: HT BFVbta Riems; calves 3 and 4: wt BFVbta Riems) and the two mock-infected calves (calves 5 and 6).

None of the animals developed specific disease symptoms or were otherwise obviously affected during the course of the study. The development of sero-reactivity to the BFVbta Gag antigen and the determination of BFVbta DNA viral load were used to investigate the course of the BFVbta infections in the calves inoculated with the two different BFVbta Riems variants. Sera from the BFVbta-infected and mock-infected calves were tested using a generic BFVbta GST-ELISA for antibodies against BFVbta Gag [6]. In all the BFVbta-infected calves, Gag-specific antibodies were detected two weeks post-inoculation (wk p.i.) (Figure 1), increased rapidly during the first four wk p.i., and were maintained at high levels until 16 wk p.i., as has been observed in cows naturally and experimentally infected with BFVbta [6,12]. The levels of sero-reactivity in all the animals were comparable throughout the whole experiment.

**Figure 2:**
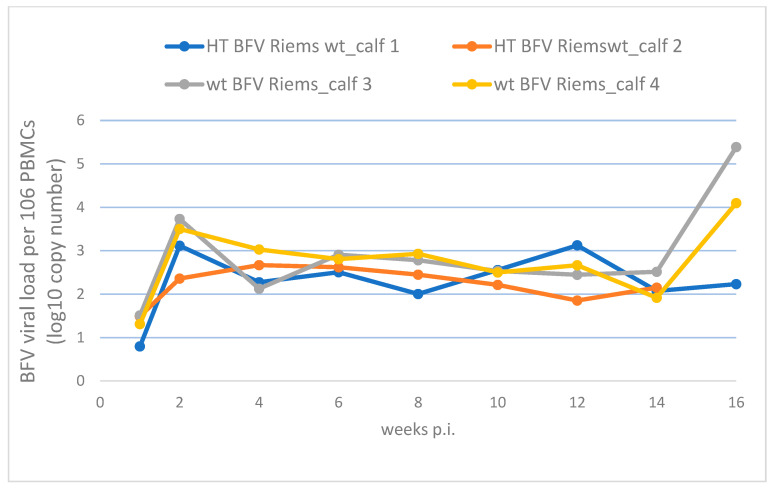
BFVbta viral DNA load in the PBMCs of calves determined during the 16 weeks following inoculation with the different BFVbta Riems variants (see legend). The BFVbta DNA load was determined using qPCR on the PBMCs of the four calves experimentally inoculated with the BFVbta variants during the first 16 wk p.i. No BFVbta DNA was detected in the mock-infected calves. The DNA of calf 2 was not available from 16 wk p.i.

Using the BFVbta-specific qPCR, DNA was detected at very low levels in the PBMCs from all the BFVbta-inoculated animals at 1 wk p.i. (Figure 2). At 2 wk p.i., the DNA levels doubled in both groups and decreased slightly after 4 wk p.i. Between 14 and 16 wk p.i., the DNA levels in both of the wt BFVbta Riems animals more than doubled, while it remained unchanged in one of the HT BFVbta Riems animals. Unfortunately, DNA from the other HT BFVbta Riems animal was not available (Figure 2).

**Figure 3:**
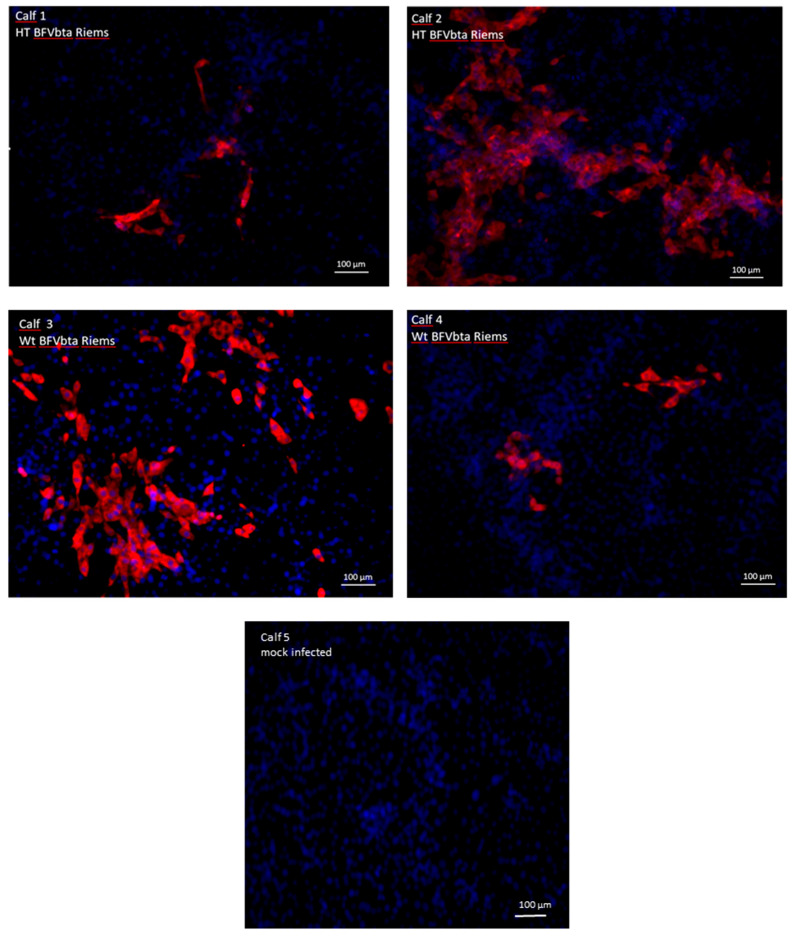
Detection of BFVbta Gag protein in co-cultures using indirect immunofluorescence. The re-isolation of the virus from the PBLs of the inoculated calves was carried out via co-cultivation with permissive MDBK cells. Indirect immunofluorescence was performed after the 3rd passage using rabbit anti-BFVbta-Gag MA polyclonal serum and anti-rabbit IgG-Alexa 595 (red) as a secondary antibody, while the nuclei were stained with Hoechst 33342 (blue).

BFVbta re-isolation from the infected calves was carried out via the co-cultivation of peripheral blood leukocytes (PBLs) with permissive MDBK cells at the end of the experiment (week 16). Co-cultures were maintained for 24 h and then passaged three times. BFVbta-specific signals, indicative of the presence of replication-competent BFVbta, were observed after the third passage in all the co-cultures using an IIF test with BFVbta Gag MA-specific rabbit antiserum (Figure 3). In both groups, the co-cultures from each of the two animals showed different rates of infected cells, stained in red, while the mock-infected control animals scored negative in the IIF test.

### 3.2. Analysis of Compensatory Mutations in BFVbta Gag Gene

Adaptive changes in the BFV Gag and Env proteins have been shown to be responsible for and/or associated with the HT phenotype following *in vitro* selection and evolution [1,10,11]. This is in line with the fact that both proteins comprise the foamy- and retroviral budding machinery [2,18]. To detect adaptive changes in the wt and HT BFVbta variants following their growth in the calves, the BFV *gag* gene was amplified from the leukocytes of the four calves at 2 wk p.i. and from the HT BFVbta Riems inoculum used for the infection of calves no. 1 and 2. The translation of the nucleotide sequences revealed that the Gag amino acid sequence of the inoculum (HT inoculum) corresponded almost perfectly with the Gag sequences from the HT BFVbta Riems pBFV-CMV-MDBK24HT parental clone (Supplementary Figure S1). Many of the HT phenotype-associated adaptive mutations were absent in both of the HT BFVbta Riems-infected animals (calves 1 and 2), with two major exceptions: firstly, the HT phenotype-associated A/T at residue 207 (A207T) was present in one out of the eight calf-derived sequences, and, secondly, seven out of the eight animal-derived sequences showed an N431S exchange. When aligning the primary nucleotide sequences, it was surprising to see that both the HT phenotype-associated adaptive changes in the Gag protein sequence and the synonymous, silent changes in the HT BFVbta Riems genome had mostly reverted back to the wt BFVbta Riems sequence in the experimentally infected calves (Supplementary Figure S2).

**Figure 4:**
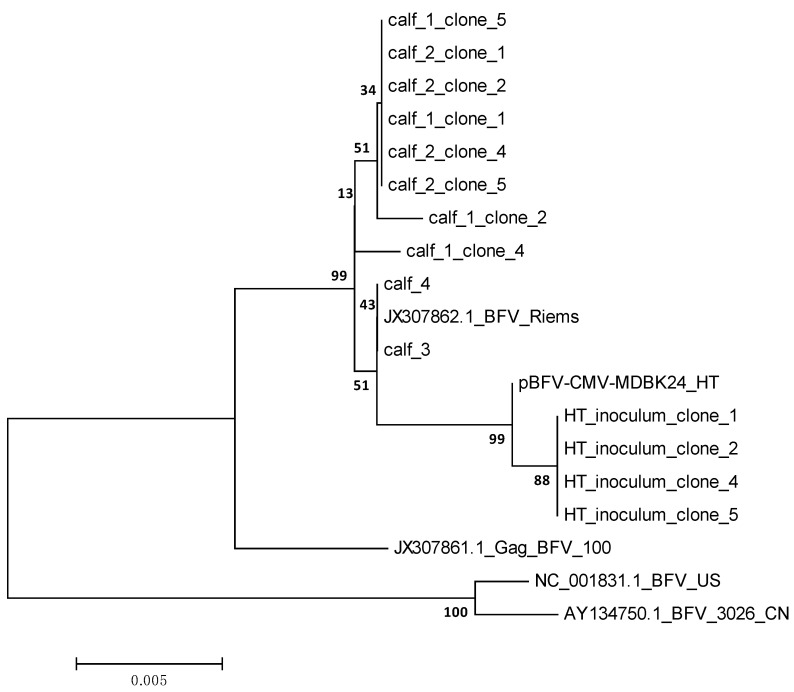
Phylogenetic tree inferred from the 1295 bp nucleotide sequence of the BFVbta Gag amplified from the four calves 2 wk p.i. and the HT inoculum used for the inoculation of calves no. 1 and 2. The analyses were conducted in MEGA 6 using the maximum likelihood method based on the Kimura 2-parameter model. The percentage of trees in which the associated taxa clustered together is shown next to the branches. The tree is drawn to scale, with branch lengths measured in the number of substitutions per site. The reference sequences were as follows: pBFV-CMV-MDBK24_HT [17]; GenBank derived: JX307862.1_BFV Riems; JX307861.1_BFV100; NC001831.1_BFV_US; AY134750.1 BFV_3026_CN.

Phylogenetic analyses showed that the Gag nucleotide sequences from these calves clustered together with sequences derived from wt BFVbta Riems, but not with sequences derived from the original inoculum (HT inoculum, i.e., HT BFVbta Riems) or the parental plasmid (Figure 4). Interestingly, the sequences derived from calves no. 3 and 4 infected with wt BFV Riems showed 100% similarity to the wt BFVbta Riems reference sequence (JX307862.1).

### 3.3. Expression of Innate Immunity Genes in Calves after Inoculation with Different BFVbta Variants

Upon confirmation in our small-scale pilot study that the parental and cloned BFVbta Riems genomes, as well as the HT cell-free variant were—at least initially—replication competent in outbred calves, we analyzed whether the outline of the study would enable the identification of BFVbta- and variant-specific effects on host cell gene expression. The impact of BFVbta infection on innate and adaptive immunity was measured via expression profiling of innate and adaptive immunity-related genes in the PBMCs of the calves shortly after infection at 1 and 3 days p.i.

**Figure 5:**
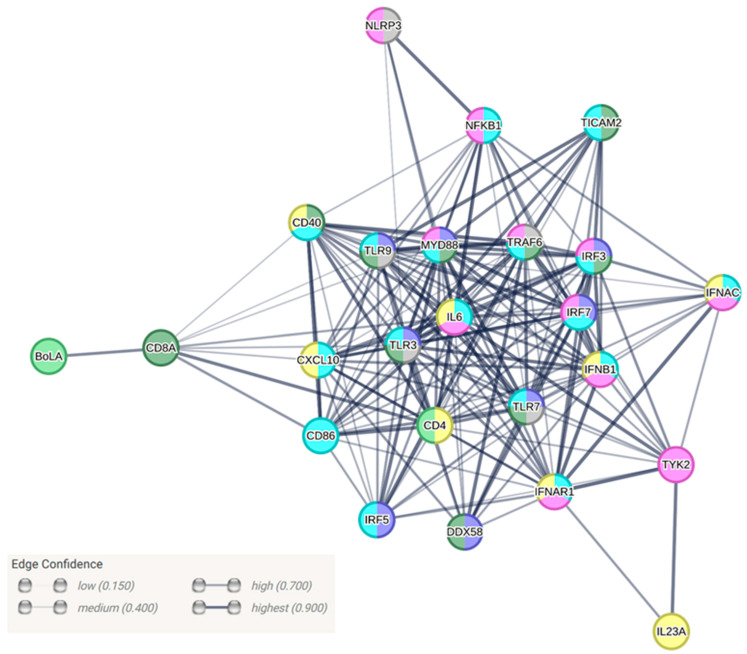
Protein interaction network created using STRING database based on *Bos taurus* genome. Edges represent protein–protein associations, line thickness indicates the strength of the supporting data, network nodes represent proteins, and colors indicate their functional categories: GO database—dark blue—GO:0032481 “Positive regulation of type I interferon production” (FDR = 9.69 × 10^−13^); green—GO:0002764 “Immune response-regulating signaling pathway” (FDR = 9.76 × 10^−12^); grey—GO:1901222 “Regulation of NIK/NF-kappaB signaling” (FDR = 3.33 × 10^−6^); KEEG-pathways—light blue—bta04620 “Toll-like receptor signaling pathway” (FDR = 1.53 × 10^−32^); pink—bta04621 “NOD-like receptor signaling pathway” (FDR = 3.48 × 10^−16^); yellow—bta04060 “Cytokine–cytokine receptor interaction (FDR = 8.74 × 10^−9^); light green—bta04612 “Antigen processing and presentation (FDR = 0.0187).

Changes in the expression of 24 bovine genes involved in the antiviral immune response were determined using a generic RT-qPCR system by comparing the infected animal groups to the uninfected controls (Table 1). More changes in gene expression were observed during the very early stage of infection (1 day p.i.), when the innate immunity mechanisms are preferentially involved, and these changes were generally stronger. At 1 day p.i., the highest changes in the expression of the tested genes were observed in the infected animals. The most significantly upregulated genes were CXCL10, IRF7, and RIG-I, while CD8A was observed to be downregulated in all the groups of infected animals 1 day p.i. (Table 1). Up-regulation of TLR7 was only observed at 1 day p.i. At 3 days p.i., genes such as RIG-I and IRF7 were still found to be upregulated in all the tested BFVbta-infected animals, though at lower levels, while CXCL10 was upregulated only in the parental BFVbta Riems-inoculated calves (Table 1). Strikingly, TLR9 was upregulated in all of the animal groups, while TLR7 no longer showed significant changes in expression at this time (3 days p.i.). However, variations between the groups were clearly observed in the different levels of RIG-I expression, which is one of the cytoplasmic sentinels for intracellular viral RNA produced during infection. A similar effect was seen for TLR7, a member of the Toll-like pattern recognition receptors. The complexity of the interactions among the proteins encoded by the tested genes were visualized using a STRING analysis, which included annotations concerning their functional categories (Figure 5) [25]. The lower induction of genes involved in the recognition of viruses by the innate immune system suggests a decrease in the replication competence of the HT BFVbta Riems variant at the very beginning of the infection.

### 3.4. Determination of IL-6 Concentrations

**Figure 6:**
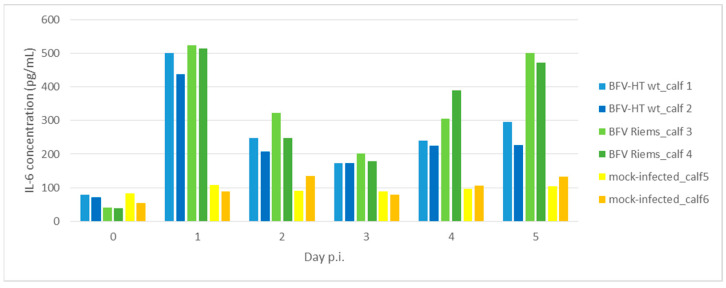
Interleukin 6 concentration in the plasma of calves infected with different BFVbta variants during first 5 days p.i.

The concentrations of interleukin 6 (IL-6) in the plasma of the calves in each of the BFVbta-infected groups as well as those in the control group were tested before the inoculation of the calves and on each of the first 5 days p.i. Significantly elevated levels of IL-6 were detected on the first day after inoculation in the plasma of all the infected calves. During days 2 to 4, the concentration of IL-6 decreased to varying degrees in all the calves, but increased again at day 5 p.i., especially in the calves infected with wt BFVbta Riems. Interestingly, throughout the whole duration of the experiment, the highest levels of IL-6 were noted in the wt BFVbta Riems-infected group (Figure 6), while the mock-infected calves which have received uninfected MDBK cells and the corresponding medium showed almost unchanged basal IL-6 levels.

### 3.5. Determination of Type I Interferon Concentrations

The concentrations of interferon alpha (IFN-α) and interferon beta (IFN-β) in the plasma of the calves in each of the BFVbta-infected groups as well as those in the control group were determined before inoculation of the calves and on each of the first 5 days p.i.

**Figure 7:**
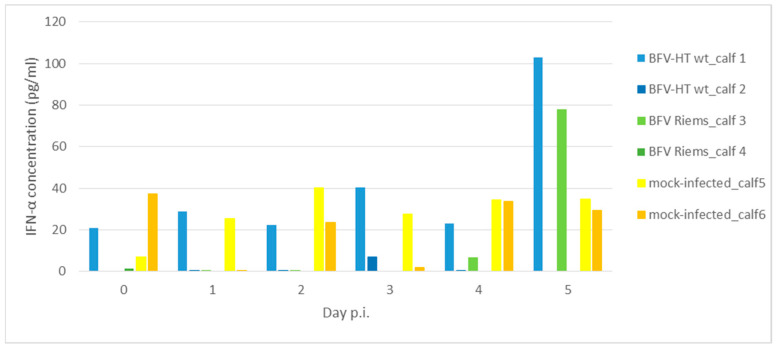
Interferon alpha concentrations in the plasma of calves infected with different BFVbta variants during first 5 days p.i.

The detection of interferon secretions was strongly limited by the sensitivity of the tests available for bovine interferons. The concentrations of IFN-α were determined using only a chemiluminescent immunoassay, and not a conventional ELISA. Despite this, IFN-α concentrations were very low in the BFVbta-infected animals throughout the duration of the study, and they were often even lower than the concentrations observed in the control animals. They also varied between animals at different time points, including day 0. The IFN-α level was consistently elevated, and the highest level was observed in animal no. 1 (which was infected with the HT variant) between days 0 and 2. It increased to almost 40 pg/mL on day 3 p.i., and to 103 pg/mL on day 5 p.i. By contrast, the IFN-α concentration in the plasma of animal no. 2 from the same group was very low throughout the whole test period, and was even undetectable on day 5 p.i. In the group infected with the wild type variant of BFVbta Riems, very low levels of IFN-α were noted during the first few days p.i. in animal no. 5, but it started to increase on day 4 p.i. and reached almost 80 pg/mL on day 5 p.i. Surprisingly, IFN-α was undetectable in calf no. 6 (which was from the same group as calf no. 5) throughout the test period (Figure 7). Unfortunately, IFN-β was undetectable in any of the animals.

## 4. Discussion

The primary aim of this small-scale pilot study on animals was to determine whether the cloned wt BFVbta Riems genome and the HT BFVbta variant derived from it would be replication competent in immunocompetent calves [17]. We also wanted to study the immune response of the bovine host towards infection with different cloned BFVbta variants. For this purpose, some of the calves were inoculated with BFVbta Riems-infected MDBK cells that were similar to the Polish BFV100 isolate used by others to infect calves, otherwise the parental and highly cell-associated BFVbta Riems isolate could not have been included for comparison with the cell-free HT BFVbta variant [12].

The data clearly show that the parental wt BFVbta Riems isolate and the HT variant, both initially and at later time points (up to week 14), robustly and almost comparably replicated in the experimentally inoculated calves. BFV100 replicated in a similar manner in a previous study [12]. However, at week 2, when the HT BFV Riems genomes of both calves were analyzed via DNA sequencing of Gag-derived amplicons, significant changes—mostly reversions to the parental BFVbta Riems genotype—were detected, as will be discussed below. Whether the increased presence of BFVbta Riems DNA at week 16 is indicative of a true biological difference—for instance differences in immune recognition or escape—is currently unknown. It might in fact reflect the better replication capacity of the wt BFVbta Riems isolate already indicated by the increased DNA counts observed until 14 weeks p.i. based on the proviral DNA scores at each time point. However, this observation is based on only three animals (see Figure 2).

In order to check whether the HT BFV variant underwent any genetic adaptations in the infected calves, BFV Gag sequences from all the calves (and from the inoculum) were analyzed at the second week post-inoculation. Gag, which is one component of the FV particle assembly and release machinery [18], was selected as it showed the most mutations during the process of BFVbta HT variant adaptation in the cell culture [1]. Interestingly, the BFV HT variant appeared to show a very strong bias towards synonymous and non-synonymous nucleotide changes, leading to a reversion to the wild type. It seems that such adaptations of the HT BFVbta variant during the *in vivo* replication in the calves was necessary for the establishment of a productive infection. One might assume that, in comparison with an infection by the highly cell-associated original wt BFVbta Riems isolate, the cell-free spread of the HT BFVbta variant would be attenuated or compromised in the immuno-competent, outbred calves. Alternatively, the HT BFVbta Riems variant, or correspondingly infected cells, could be an easier target for the host immune system. In either scenario, the HT BFVbta Riems variant lost its cell culture-acquired adaptations and almost completely reverted back to the wt, cell-associated genotype and phenotype that had been established over a long period of BFVbta coevolution with its authentic bovine host [2,26]. Interestingly the virus underwent the adaptation process within just two weeks after inoculation, i.e., before viremia and full seroconversion.

Although the shift to the wt genotype was comprehensive, we detected examples where the reversion was not complete and where additional changes occurred in the animals. The HT phenotype-associated A/T at residue 207 (A207T) was present in one out of the eight calf-derived sequences. In addition, seven out of the eight animal-derived sequences showed an N431S exchange, and one had the wt BFVbta Riems sequence. Whether and how these changes contribute to viability in calves remains to be clarified. Attempts to study any corresponding calf-adapted changes or reversions in the HT BFVbta Env for were not possible since the PCR-mediated amplifications of full-length *env* sequences were not successful, possibly due to the much larger amplicon size. Since the functional genetic reversion was so fast, and since it affected almost all the synonymous and non-synonymous changes in the HT BFVbta Riems variant, one might also consider other causes for this observation. Although we analyzed the HT BFVbta Riems inoculum using low-coverage sequencing, the virus stock might have been contaminated with miniscule amounts of wt BFVbta Riems. A more sensitive PCR-based analysis, however, did also not detect any wt BFVbta Riems sequences. Considering the difficulty of detecting rare variants, one could still imagine that traces of wt BFVbta Riems outcompeted the bulk HT BFVbta Riems due to differences in their viability and replication competence in outbred and immune-competent animals. In spite of such a possibility, we may nonetheless clearly conclude that the cell culture-adapted HT BFVbta Riems variant is not as well adapted for growth in animals as the wt BFVbta Riems. This supports the generally accepted notion that cell culture-derived viruses—and especially engineered and *in vitro*-selected variants thereof—display reduced viability in immunocompetent, outbred hosts [27]. In addition, the data indicate that even synonymous nucleotide exchanges selected *in vitro* in bovine immortalized MDBK cells undergo negative selection in calves, suggesting that cis-acting elements are also of importance for efficient replication in an animal host.

In order to check the host–virus interactions, we analyzed the expression of selected innate and adaptive immune genes very early in the BFVbta-infected calves, i.e., before the DNA load was detected and before compensatory mutations were noted. The analyses showed the consistent activation or repression of defined genes in the wt and HT BFV Riems-infected animals at both 1 day p.i. and 3 days p.i. In all of the infected animals, the chemokine CXCL10 was very strongly upregulated at 1 day p.i., but not at 3 days p.i.; at this point, only the wt BFVbta Riems-infected animals showed slightly increased expression of CXCL10. The RIG-I and IRF7 genes were clearly upregulated at 1 day p.i. in all the BFVbta-infected animals (9.69- to 18.4-fold), while at 3 days p.i., an attenuated increase was (still) detectable (3.13- to 6.8-fold). In contrast, TLR9 was only upregulated in all of the animals at 3 days p.i. (2.55- to 2.730-fold). The only consistently downregulated gene was CD8A at 1 day p.i. (0.35- to 0.15-fold), while TLR8 was downregulated at a steady level of 0.42 at both time points in the HT BFVbta animals and at 3 days p.i. in the wt BFVbta Riems group, and its expression was almost comparable with that observed in the control group at 1 day p.i. The expression profile determined early in the BFVbta-infected calves was consistent with the induction of an innate immune sensing and a mounting response towards the infection by the BFVbta-infected calves that was absent in the calves inoculated with the non-infected bovine MDBK cells.

In order to check the downstream effects of the innate immune sensing-mediated signaling cascade, the concentrations of IL-6 and IFN-α were measured [28,29] during the first 5 days p.i., but they did not exactly reflect the transcriptional profile of the PBMCs. This observation may be related to the fact that additional, non-PBMC cell populations contributed to the protein levels measured here. Similarly, local, individual cell populations may also have contributed to the highly variable IFN-α protein levels detected in the animals very early after infection and the tendency of the IFN-α levels to increase at later time points (Figure 7). However, the variations observed in the IFN-α levels in each group may also have been a consequence of individual, host-related immune responsiveness. The low-level fluctuations in IL-6 expression and the lack of a consistent induction of IFN-α in the animals infected with the mock-treated MDBK cells indicate that the observed changes in the innate immune genes detected in the BFVbta-infected calves was not a consequence of using infected cells as an inoculum. This observation may even indicate that the utilization of BFVbta-infected bovine MDBK cells is superior to the utilization of infected canine Cf2Th cells, which have been used in previous studies. However, we did not analyze the innate immune responses this time [12].

Since the inoculation of BFVbta-infected bovine MDBK cells resulted in strong signs of cytopathic effect compared with the non-infected control cells without cell pathology, a certain amount of the innate host immune response may have been due to the effectors released by the infected/dying BFVbta-infected cells and thus only indirectly related to the initial BFVbta replication in the calves. In this way, the massive transfer of viral components via inoculation of the immune-competent host with BFVbta-infected and severely compromised/damaged cells could have contributed to the triggering of innate sensing in order to strongly activate the antiviral host immune system, as was detected here (Table 1).

Following this idea, the viral components released from the BFVbta-infected cells used as inoculum to compare the cell-free and cell-to-cell transmission of BFVbta Riems variants together with the ongoing BFVbta replication and gene expression should have stimulated innate sensing to different degrees during the very first days after infection. The number of infected cells was comparable in all the BFVbta inoculations and, thus, the input inoculum—and not the starting BFVbta replication—may have been the strongest innate immunity trigger at day 1, as was exemplified by the early induction of the dsRNA pattern-recognition receptor RIG-I, the ssRNA sensor TLR7, the downstream interferon regulatory factor 7 (IRF7), and the downstream cytokine CXCL10. At day 3, the flooding of the calves’ immune systems with BFVbta-infected and non-infected cells may no longer have played a major role, leading to an overall decline in signal intensities and to a shift to another innate immune sensing signature that may have been determined by a spreading virus infection in a limited number of cells. For example, TLR9, which may also detect DNA/RNA hybrids generated as reverse transcription intermediates, was activated in all the BFVbta-infected animals at 3 days p.i. [30,31].

There are a limited number of reports on the innate immune sensing of foamy viruses which describe the immune response following the infection of human cells with simian foamy virus (SFV) [29] or prototype foamy virus (PFV) [32]. Plasmacytoid dendritic cells were shown to produce type I IFN upon incubation with SFV virions or SFV-infected cells and to detect FV RNA via the TLR7-mediated pathway [29]; this was confirmed via the up-regulation of TLR7gene. Furthermore, in human myieloid cells, PFV pathogen-associated molecular patterns were sensed using the DNA sensor cyclic GAMP synthase (cGAS), and this led to the mounting of an interferon regulatory factor 3-(IRF3)-dependent interferon-stimulated gene (ISG) response [33].

In the present study, the overexpression of TLR7, RIG-I, and TLR9 suggests the involvement of these pattern-recognition receptors in the innate immunity sensing of BFVbta, but the low-level IFN-α response detected here was unexpected. IFN-α, as a type I IFN, is known for its ability to directly induce an antiviral response within infected cells and surrounding cells through the upregulation of genes that can antagonize virus replication [33]. The low concentration of type I IFN can be explained by the fact that it is mainly produced by dendritic cells, which represent less than 1% of PBMCs [21]. Alternatively, the low amount of somewhat inconsistent data concerning IFN-α in the early stage after infection could be a result of currently unknown BFVbta immune evasion mechanisms or stochastic events in this small-scale study.

## 5. Conclusions

In summary, we have demonstrated that both cell-associated and *in vitro*-selected high-titer and cell-free transmission BFVbta variants are able to establish productive infections in calves. However, compensatory mutations were observed in the *gag* gene of the CF HT BFVbta variant soon after infection, and these might cause slight variations in the expression of genes involved in the innate immune sensing of the host cells, though presumably they allowed virus to replicate more efficiently. Thus, the animal model shown here, together with the availability of defined BFVbta isolates, will facilitate studies concerning host–virus (co)evolution and virus–host interactions in authentic, manageable, and meaningful animal models.

## Figures and Tables

**Table 1:** Relative changes in gene expression in PBMCs of experimental animals at 1 and 3 days p.i.

	Gene Symbol	Fold Change (Compared with Mock-Infected Control Group)			
		HT BFVbta Riems Group 1		wt BFVbta Riems Group 2	
		1 Day p.i.	3 Days p.i.	1 Day p.i.	3 Days p.i.
Innate Immune Response	TLR3	1.23	0.77	1.40	1.55
	TLR7	**2.04**	0.99	3.35 *	0.63
	TLR8	0.42 *	**0.42**	1.15	**0.42**
	TLR9	0.77	**2.73**	**0.42**	**2.55**
	RIG-I (DDX58)	9.69 *	**3.13**	11.63 *	**3.28**
	NLRP3	1.13	1.00	2.09 *	1.32
	IRF3	1.50 *	0.91	2.40 *	0.84
	IRF7	10.92 *	3.70 *	18.38 *	6.81 *
	MYD88	0.78	0.49 *	1.13	0.86
	NFKB1	1.16	1.20	1.60 *	0.96
	TICAM1	1.10	0.74	0.86	1.12
	TRAF6	1.09	0.78	1.32	0.99
	BOLA	1.90 *	1.62 *	1.86 *	2.02 *
	TYK2	0.44 *	0.43 *	0.54 *	0.65
	IFNAR1	1.47 *	1.20	1.30	0.73
	IFNAC	1.47	1.15	1.86 *	1.36
	IFNB1	0.83	0.86	**2.13**	0.79
	CD8A	0.35 *	0.60	0.15 *	0.82
	CD4	0.64 *	**0.47**	0.29 *	1.02
Innate/Adaptive Immune Response	CD40	1.93 *	1.05	1.85 *	0.78
	CD86	1.40	0.56	1.75 *	1.20
	CXCL10	35.09 *	0.92	76.78 *	**2.53**
	IL6	1.77 *	**2.05**	**4.21**	1.47
	IL23A	1.74 *	1.06	1.24	1.09

Relative changes in gene expression tested in PBMCs of experimental animals from particular groups at 1 day p.i., shown as fold-changes calculated using the ΔΔCt method. Fold-change values greater than 2 and less than 0.5 are indicated in bold red and bold blue, respectively. The *p*-values are calculated based on a Student’s *t*-test of the replicate 2^−ΔCt^ values for each gene in the control group and the infected groups, and *p*-values less than 0.05 are indicated with an asterisk.

## Data Availability

Not applicable.

## Supplementary Materials

The following supporting information can be downloaded at: https://www.mdpi.com/article/10.3390/v15081772/s1, Figure S1:Alignment of 431 aa Gag protein sequences translated from sequences amplified from calves two weeks post inoculation with HT BFVbta Riems (calves 1 and 2) and wt BFVbta Riems (calves 3 and 4) variants and HT BFVbta Riems inoculum (HT inoculum) used for inoculation of calves 1 and 2; and Figure S2: Alignment of 1295 bp nucleotide sequence of BFVbta gag gene amplified from calves two weeks post inoculation with HT BFVbta Riems (calves 1 and 2) and wt BFVbta Riems (calves 3 and 4) variants as well as HT BFVbta Riems inoculum (HT inoculum) used for inoculation of calves 1 and 2.
