# Supplementary material for: An Outbred Calf Model for Determining Innate Immune Sensing and Evolutionary Trajectories of a Cell Culture-Adapted Bovine Foamy Virus Variant
Source: Viruses. 2023 Aug 20;15(8):1772. doi: 10.3390/v15081772 (PMC10458543; doi:10.3390/v15081772)
Supplement: Supplementary file 1 [file viruses-15-01772-s001.zip › Supplementary files/Supplementary file Figure S1.pdf]

Figure S1. Alignment of 431 aa Gag protein sequences translated from sequences amplified from calves two weeks post inoculation with HT BFVbta Riems (calves 1 and 2) and wt BFVbta Riems (calves 3 and 4) variants and HT BFVbta Riems inoculum (HT inoculum) used for inoculation of calves 1 and 2. Red boxes indicate the sites where adaptive mutations in HT BFVbta infected calves were noted. Dark blue bars indicate insertions and gray bars deletions leading to frame-shift mutations. Reference sequences were as follows: pBFV-CMV-MDBK24_HT [1] and GenBank derived: JX307862.1_BFV Riems ; JX307861.1_BFV100; NC001831.1_BFV_US ; AY134750.1 BFV_3026_CN.

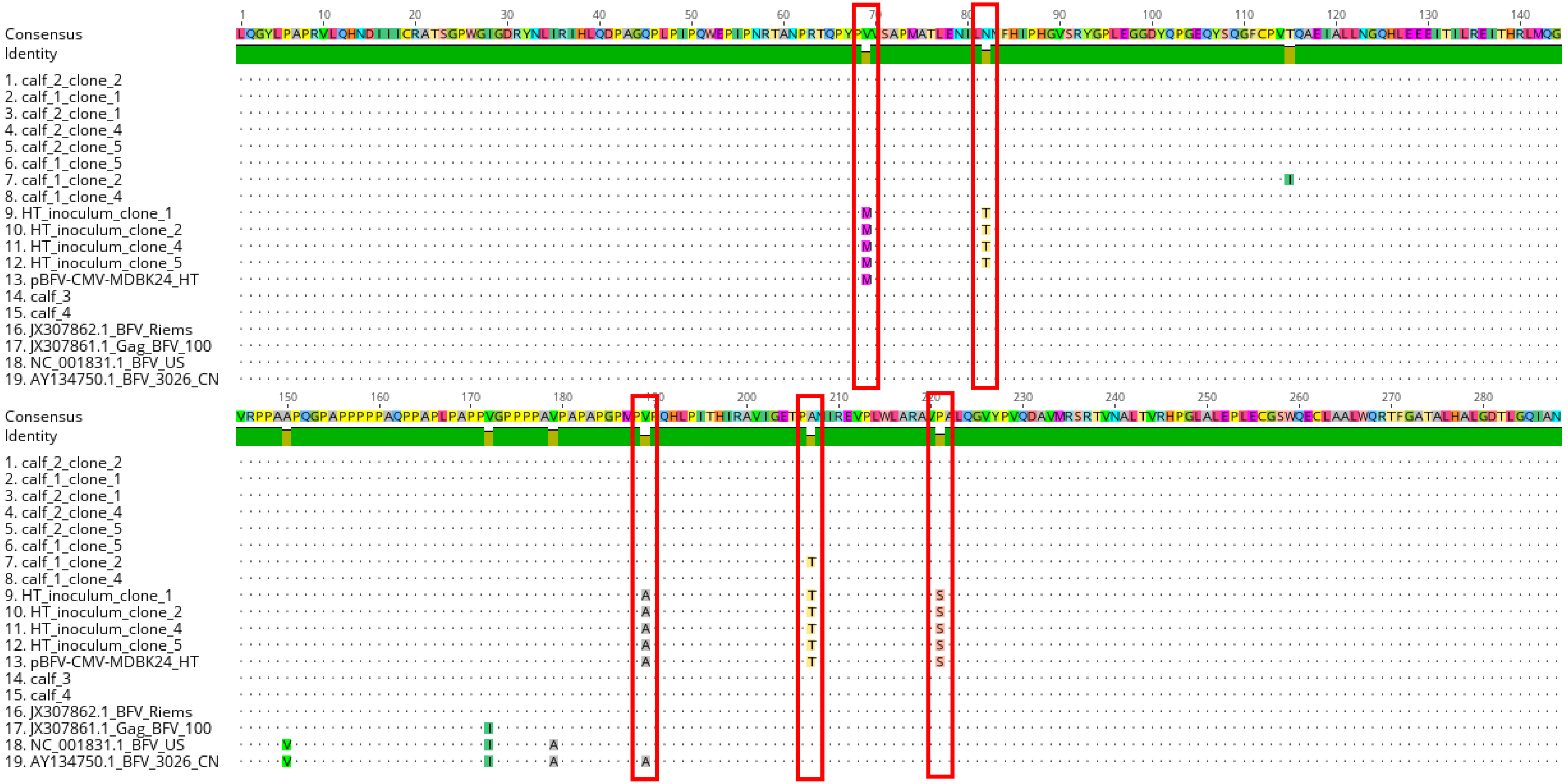

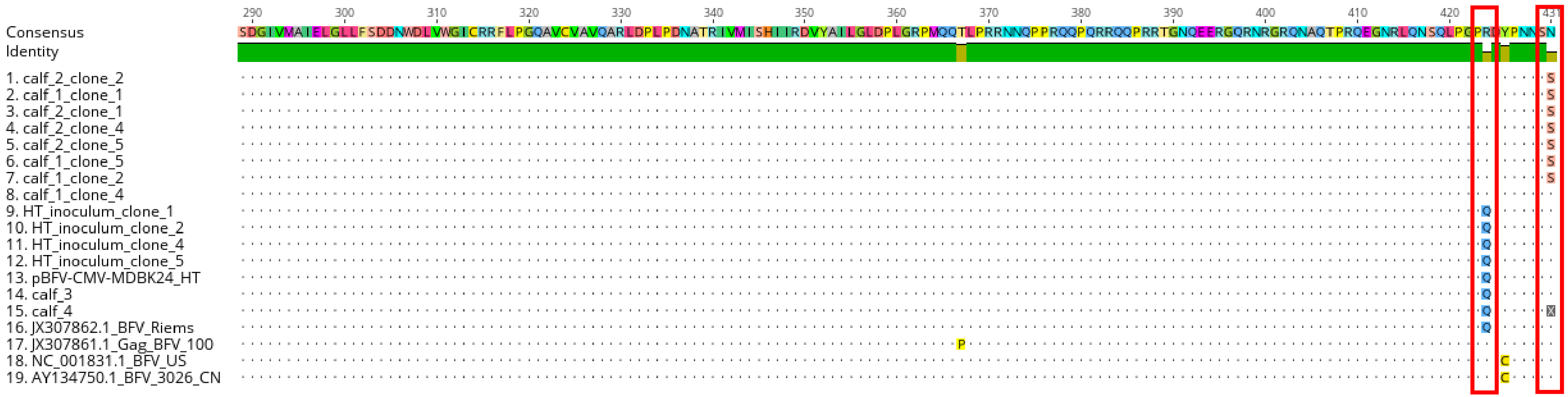

290
300
310
320
330
340
350
360
370
380
390
400
410
420
431
Consensus
Identity
1. calf_2_clone_2
2. calf_1_clone_1
3. calf_2_clone_1
4. calf_2_clone_4
5. calf_2_clone_5
6. calf_1_clone_5
7. calf_1_clone_2
8. calf_1_clone_4
9. HT_inoculum_clone_1
10. HT_inoculum_clone_2
11. HT_inoculum_clone_4
12. HT_inoculum_clone_5
13. pBFV-CMV-MDBK24_HT
14. calf_3
15. calf_4
16. JX307862.1_BFV_Riems
17. JX307861.1_Gag_BFV_100
18. NC_001831.1_BFV_US
19. AY134750.1_BFV_3026_CN
